# The Role of Advanced Sensing in Smart Cities

**DOI:** 10.3390/s130100393

**Published:** 2012-12-27

**Authors:** Gerhard P. Hancke, Bruno de Carvalho e Silva, Gerhard P. Hancke

**Affiliations:** 1 Advanced Sensor Networks Research Group, Department of Electrical, Electronic and Computer Engineering, University of Pretoria, Pretoria 0002, South Africa; E-Mails: silvabjc@gmail.com (B.C.S.); gerhard.hancke@rhul.ac.uk (G.P.H.); 2 Information Security Group, Royal Holloway, University of London, Egham TW20 0EX, UK

**Keywords:** advanced sensing, sensor networks, smart cities, internet of things

## Abstract

In a world where resources are scarce and urban areas consume the vast majority of these resources, it is vital to make cities greener and more sustainable. Advanced systems to improve and automate processes within a city will play a leading role in smart cities. From smart design of buildings, which capture rain water for later use, to intelligent control systems, which can monitor infrastructures autonomously, the possible improvements enabled by sensing technologies are immense. Ubiquitous sensing poses numerous challenges, which are of a technological or social nature. This paper presents an overview of the state of the art with regards to sensing in smart cities. Topics include sensing applications in smart cities, sensing platforms and technical challenges associated with these technologies. In an effort to provide a holistic view of how sensing technologies play a role in smart cities, a range of applications and technical challenges associated with these applications are discussed. As some of these applications and technologies belong to different disciplines, the material presented in this paper attempts to bridge these to provide a broad overview, which can be of help to researchers and developers in understanding how advanced sensing can play a role in smart cities.

## 1. Introduction

**Figure 1.**
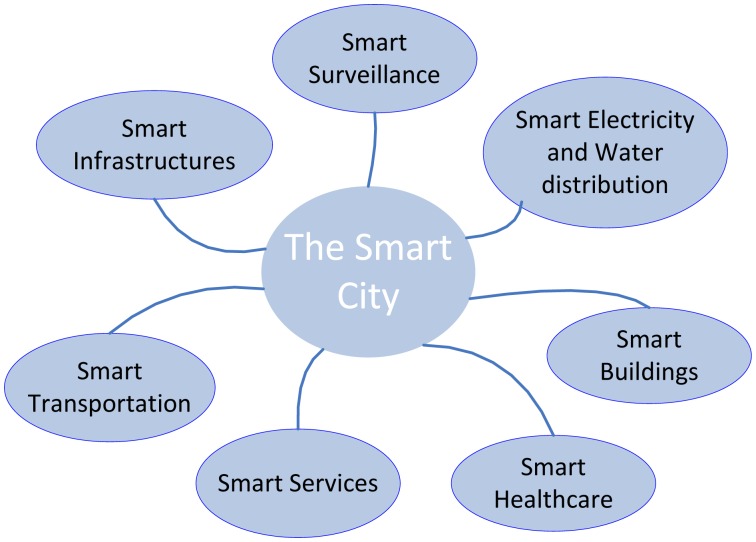
Sensing in smart cities.

A smart city is a city which functions in a sustainable and intelligent way, by integrating all its infrastructures and services into a cohesive whole and using intelligent devices for monitoring and control, to ensure sustainability and efficiency [1]. In a world where population numbers are constantly rising, significantly driving the consumption of resources causing resource shortages and climate change, the incentive for innovative solutions is evident. Urban areas, in particular, are responsible for the major part of resource consumption, instigating an increasing need to create smarter infrastructures, in search of greener and more energy efficient urban dynamics. Solutions to these issues comprise of improvements to a majority of components of urban dynamics, as illustrated in Figure 1.

It is predicted that the global economy will be significantly disproportionate due the growth of cities, with forecasts that by 2050 more than 6 billion people will live in urban areas [2]. This growth will aggravate further existing energy and climate related challenges. To solve these challenges, cities which are more resource efficient and technology driven, are necessary.

Sensing is at the heart of smart infrastructures, which can monitor themselves and act on their own intelligently. Using sensors to monitor public infrastructures, such as bridges, roads and buildings, provides awareness that enables a more efficient use of resources, based on the data collected by these sensors. Real-time monitoring eliminates the need for regular scheduled inspections, therefore reducing costs; measuring energy consumption in households allows for accurate load forecasting; and sensors deployed in roads for traffic monitoring collect data which is necessary for the implementation of intelligent transportation systems (ITS).

For these approaches to be effective, sensors have to be deployed in very large numbers and they have to be interconnected, so that the collected data can be sent to a central information system, where intelligent decisions based on this data can be made. This poses a number of challenges. Firstly, there needs to be a communication infrastructure in place for these sensors to communicate with each other. Secondly, what is the most efficient way to aggregate and process this data?

Aspects related to sensing in smart cities as well as efficient processing of data collected by these sensors is discussed in the remainder of this paper. It is organized as follows: Sections 2 and 3 review the current state of sensing and enabling technologies, respectively. In Section 4 existing sensing platforms for smart cities are discussed, and in Section 5 sensing applications in smart cities are discussed. Some ongoing and future pilot projects for smart cities are described in Section 6. Challenges and future directions are discussed in Section 7, and the paper is concluded in Section 8.

## 2. The Sensing Evolution

Sensors are a crucial component of any intelligent control system. A process is improved based on its environment and for a control system to be aware of its environment, it is typically fitted with an array of sensors, from which it collects the required data. It then uses the appropriate variables to characterize its environment and adjusts its operations accordingly.

The availability of a multitude of different sensors and continuously evolving technology enable applications that were infeasible in the past due to high costs and limited availability. Technology advances not only drive the innovation behind sensors, they also enable sufficient processing power for small-scale devices to which these sensors can be interfaced to at a relatively low cost. From the perspective of the requirements for smart cities, wide availability of these technologies translates to a large number of opportunities in terms of sensing.

As an example within the context of the smart city, meters to determine gas, electricity and water consumption have traditionally been mechanical. However, smart metering implies a new generation of technologies [3]. As with mechanical solutions, they must be robust, cheap, easy to maintain and reliable, as their readings will be used to determine billing. Electricity meter readings have evolved from the manual procedure of reading the mechanical meter, to automatic meter reading (AMR) which were deployed to reduce costs and improve the accuracy of meter readings, eventually to an advanced metering infrastructure (AMI) which differs from the AMR in that it enables two-way communications with the meter, driven by a growing understanding of the benefits of two-way interactions between system operators, consumers and their loads and resources.

A sensor is a converter which converts parameters of a physical nature to an electronic signal, which can be interpreted by humans or can be fed into an autonomous system. These signals for conventional sensors, amongst others, include light, pressure, temperature, humidity, moisture and a variety of other parameters.

More sophisticated sensors include accelerometers which can be used to measure acceleration and vibration. In the context of structural health monitoring (SHM), for instance, sensors like corrosion rate sensors (working on the principle of an increase in electrical resistivity due to corrosion); acoustic emission sensors (used to detect propagation of sound waves); and magneto-strictive sensors (detects the change in magnetic induction in the material caused by strain or stress). An even later generation of sensors is based on semiconductor physics and nano-technology, and intelligent sensing devices include, amongst others, smart phones.

CMOS based sensing, due to the relative low cost, enables a range of smart sensors. In [4], a smart temperature sensor with a one-point calibration technique and MOSFET circuits is proposed. This sensor is primarily aimed at low-power devices. Other CMOS-based sensor applications include monitoring the quality of food products, by observing the temperature and time of exposure, which are parameters related to the quality of food products [5]. Within the context of the smart city, these devices can also sense air and water quality, amongst others.

In particular, stick-on or printed sensors can be attached to devices as labels to measure various physical parameters, such as humidity [6], temperature [7] and gas [8]. Also, sensors have been proposed in plastic foil, e.g., chemical gas sensors, implemented on a variety of substrates, coated with a sensing film. These enable sensing of multiple parameters from a single sensor. For instance, on a single flexible foil, a multi-parameter sensor for temperature, humidity and gas, using flexible polyimide foils, is proposed in [9], with the capability of sensing propane and similar gases. A possible application for these sensors, as indicated in [9], is humidity monitoring, by embedding these sensors into construction materials, or environmental monitoring [10], an important capability for smart cities.

Nano-technology, although mostly applicable for bio-sensing applications, is a key enabling technology for sensor development, as advances in nano-technology will undoubtedly drive development in MEMS and photonics, inevitably leading to the development of highly sophisticated but low-cost sensors. This technology will enable applications where not only an individual's surroundings can be sensed, but also the person's health, at high granularity, for instance, and linked to social networks.

The ever increasing number of smartphones opens up a totally new sensing scenario. Smartphones are fitted with a variety of sensors such as GPS, gyroscopes, accelerometers and compasses, enabling a variety of crowd sourcing applications, which will eventually be augmented by the Internet of Things. In particular, collaborative data collection is a popular crowd sourcing application. Crowd sourcing will be discussed in Section 5.10.

## 3. Enabling Technologies

Some technical issues regarding communication between sensor nodes still have to be resolved. In the context of the Internet of Things (IoT), the communication between sensors nodes has to be wireless, as cabling costs for millions of sensors is impractical and extremely expensive. Low power communication standards, suitable for an extremely large number of devices and their heterogeneity, are necessary. In particular, depending on location and necessary coverage, there are a number of different networks in smart cities. Depending on location and coverage, these can be classified as [11]:

**Home Area Networks:** These typically use short range standards, with operating frequencies in the ISM bands. Standards include ZigBee, Dash7 and Wi-Fi (802.11 g/n), or wired standards such as Ethernet. All the monitoring and control system's components in a home are connected by the HAN.

**Wide Area Networks:** These provide for communication between utilities and customers' premises, which requires a much broader coverage than HANs, with infrastructure such as leased lines based on optic fiber or broadband wireless, e.g., 3G and LTE, and managed by service providers.

**Field Area Networks:** Typically used in a smart grid to connect customers' premises to substations.

Table 1 compares the different communication standards listed in this section.

### 3.1. ZigBee

With the advent of ubiquitous computing, one of the concepts of smart cities is to have sensors deployed virtually everywhere, in the millions, such as in PlanIT Valley, where the aim is to have 100 million sensors. As sensor nodes are typically designed to be low-power devices, the communication standard has to take that into account, as communication consumes the most energy.

Low power standards like IEEE 802.15.4 have limited range and a significant number of repeaters would have to be deployed in a city for full coverage. Additionally, addressing schemes would have to support billions of devices. 6LoWPAN defines mechanisms which allow transmission of IPv6 packets over IEEE 802.15.4 based networks. Although this certainly presents itself as a solution to the addressing issue, the coverage issue is tightly coupled to the communication technology itself, which is inherently short range (in the order of hundred meters).

### 3.2. Dash7

Dash7 is a promising standard for WSNs and is aimed at long distance, low power sensing applications. Some of these applications include building automation and logistics, to name a few. This protocol stack has a very small footprint; its communication range is in the order of kilometers; and it operates at 433 MHz, a frequency which allows better penetration than 2.4 GHz [12]. For home area networks (HANs), for instance, this frequency is appealing, as it allows better penetration through walls than 2.4 GHz. For wide area networks (WANs), Dash7 is also appealing because of the long ranges. Dash7 has received considerable attention for military applications, including substantial investments. Nevertheless, to the best of our knowledge, there is no significant active academically driven research focused on Dash7.

### 3.3. 3G and LTE

In terms of existing wireless communication infrastructures, 3G and LTE, have available infrastructures worldwide, even in third world countries. These technologies are meant for broadband connectivity, and were not designed for short range, low power operation. Although they have been adopted by mobile devices such as smartphones, these devices operate on a rechargeable battery, in contrast to sensor nodes which are ideally deployed sparsely, with no intention of charging or replacing batteries. Furthermore, these services are provided by service providers and the data cost is still relatively expensive compared to their fixed line counterparts such as ADSL. With millions of sensors uploading gigabytes or even terabytes (in case of video) of data to the cloud, these technologies are rendered infeasible for IoT. Furthermore, these standards are not optimized for use in sensor networks (*i.e.*, low power). Hence, for these technologies to be adopted for sensor networks, power consumption has to be considered. In the absence of embedding these technologies into the sensor node (*i.e.*, integrating LTE or 3G into the sensor platform), gateways can be used between the sensor devices and the web, as shown in [13]. LTE, in particular, has been a main enabler of high performance M2M applications, due to coverage, high throughput and low latency.

### 3.4. RFID and NFC

With regards to short range communication, there are two prominent technologies in the context of smart cities. Radio Frequency Identification (RFID) consists of an RFID tag (which can be either passive or active), where data is stored, and an RFID reader, which induces an electromagnetic field when in near proximity to passive tags and by this means provides power to the devices (longer ranges for active tags), enabling it to read data from the tag's memory. RFID enables a range of applications for smart cities, such as localization and tracking of objects [14], healthcare applications [15], asset management [16] and smart parking [17], to name a few. As the circuitry in tags is printed, it is natural to integrate these tags with *smart label sensors*, therefore creating RFID tags which do not only contain data which has been manually written, but they also capture environmental data as described in [9]. Hence, each tag can also act as a sensor. Additionally, because passive tags are inexpensive, these can be deployed in very large numbers for *pervasive sensing*. Furthermore, the passive nature of these tags translates to low cost and low power consumption, as these tags only need to be powered when needed.

Near Field Communication (NFC) is used in mobile and similar devices, for a very short bi-directional communication range (in contrast with RFID which is unidirectional). These ranges are usually in the centimeter range. The recent integration of NFC into smartphones has enabled a range of applications which facilitate day-to-day activities and enable smart living. A selection of general applications which have emerged with NFC is given:

The most predominant application of NFC is the digital wallet. This allows customers to use their smartphones as a substitute for their cards, which can be for public transportation, bank, access control or identification [18].

Since NFC is bidirectional, it is possible to use it to share data between devices, multimedia as well as documents, such as business cards or similar contact information [19].

Occupancy and user context in smart homes are accomplished by placing NFC tags at strategic locations in a house and have these tags interface with the central information system It is possible to determine whether a user has entered a room and to change the settings according to the location (e.g., switch on Wi-Fi if the user enters the house) [17].

The following are real-world examples of NFC application within the context of the smart city:

**Smart energy metering:** NFC-enabled post-pay electricity meters were deployed in the city of Chongqing, China, which enable consumers to read their smart meters with an NFC phone. Payment for the electricity is automatically done after the encrypted reading has been send over-the-air to the service's banking back-end [20].

**Data acquisition and control:** NFC enabled smartphones can be used to interface with control systems for remote control [21] and can be used as platforms for advanced measurement and processing [22].

**City touristic surfing:** With an NFC enabled smart phone and smart posters disseminated along the city, the user can navigate through points of interest within the city [23].

**Smart car parks:** Motorists can use their NFC enabled smart phone as an electronic ticket to enter the parking lot and when leaving as an electronic wallet to affect payment [24].

With the addition of NFC to smartphones and the widespread of smartphones, NFC is envisaged as a key enabling technology in smart cities.

## 4. Sensing Platforms

With the advent of ubiquitous sensing, various devices and platforms are currently available for this purpose. Although ideas to implement these platforms have been around for decades, only recently have these systems been feasible from a cost perspective, driven by reduced manufacturing costs as well as low-cost electronic components such as radio transceivers, microcontrollers, microprocessors and sensors. Sourcing these components in bulk becomes increasingly less expensive as technology progresses.

Sensor nodes can be interfaced to a number of sensors. The most common include humidity, light and temperatures sensors. These platforms are built in such a way that the sensed data is collected by the sensors, sometimes pre-processed, and then transmitted to a sink node via other sensor nodes; hence they are usually set up in a network, also known as a wireless sensor network (WSN).

Additionally, these systems will be deployed outdoors and thus have to cope with the harsh environmental conditions. In this context, properties from industrial control networks can be applied in these sensor networks for added robustness and reliability [25].

### 4.1. Wireless Sensor Networks

**Figure 2.**
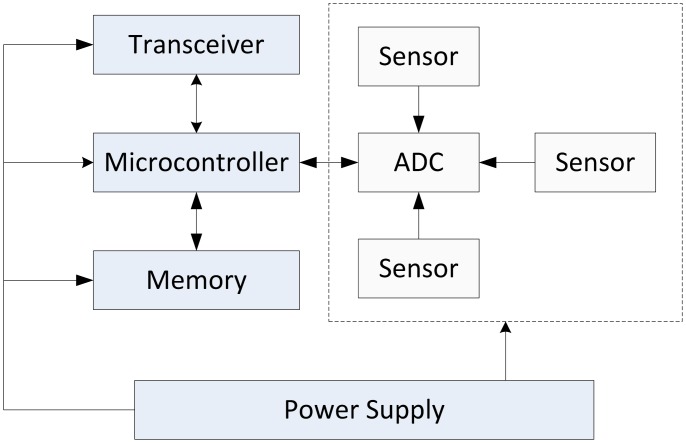
Architecture of a wireless sensor node.

Wireless sensor networks consist of wireless sensor nodes, which are devices equipped with a processor, a radio interface, an analog-to-digital converter, multiple sensors, memory and a power supply. The general architecture of a wireless sensor node is illustrated in Figure 2.

A range of open source sensor nodes are available to the research community. Some of the most widely used sensor nodes recently in research are Mica, Mica2, IMote, Telos, TelosB, Stargate and IMote 2. Table 2 contains details on available sensor motes used in research and commercial applications. The utopia of WSNs is an extremely tiny low-power, low-cost sensor node which can be deployed in any environment, with sufficient battery power to last for a few years, with the capability to continuously sense its surroundings. In reality, even with recent advances in microelectronics, these have still not been realized. The only project to come close to this idea was Smart Dust, which was active up to the early 2000s [26].

#### Energy Harvesting

Wireless sensor networks are characterized by severe resource constraints, one of which is the reliance on battery life. With the deployment of sensor nodes in very large numbers in smart cities, it is infeasible to replace or recharge batteries in these devices. Creating energy efficient solutions from protocol design to sophisticated power management schemes are efficient and necessary methods, but not sufficient. A popular way of supplementing power includes solar panels, but sunlight is not always available and it is not the most cost effective solution. The harvesting of energy from alternative sources in the environment is actively being researched and developed. Examples of these energy sources include thermal, light (solar), wind, mechanical (vibration) and many others. However, efficient ways of capturing this energy are vital, as the energy from these sources is usually available in extremely small quantities.

Hence, ways to scavenge energy from the environment to prolong the nodes' lifetimes and improve market adoption are essential. Sources are typically from light, heat and sources of kinetic energy such as vibrations, etc. Of course, this technology can also be applied to drive any other low-energy device.

Methods of energy harvesting, in the context of smart cities include:

**Wind energy:** Small scale turbine rotation is converted to electrical energy. Such turbines can be located in open places where wind is easily accessible. For instance, nodes used for structural health monitoring, deployed in bridges and similar structures, can readily use this kind of harvesting. Other methods involve the use of piezo-electric materials [27] or vibrating membranes [28].

**RF energy:** An antenna receives RF signals and an RF-DC converter module converts the RF signals to DC voltages. With multiple antennae emitting signals, this type of harvesting is worth exploring [29].

**Electric field:** Electric and magnetic fields around power lines, for instance, can be exploited for energy scavenging, as shown in [30,31]. Sensors deployed in the near proximity of these overhead lines can exploit these electric or magnetic fields.

**Vibrations and movement:** Kinetic energy, from vibrations for instance, can be harvested in many different locations. For instance, at utility's facilities, nodes deployed for condition monitoring can exploit vibrations for energy scavenging, as shown in [32]. Vibration is probably the most efficient energy sources for scavenging, as well as HVAC ducts in office buildings.

**Light and Thermal Sources:** These are common both indoors and outdoors. In houses for instance, these can be ambient artificial light or heat from appliances [33].

**Piezo-electric harvesters in bridges and highways:** To meet the demands in power requirements for sensor nodes for SHM, the structure's vibration, in case of bridges or highways, is a good energy scavenging source [34].

Although there is a lot of research and development ongoing in this domain, these techniques are still not completely mature for powering of low-power devices, and even when they are used, it should be for ultra low duty cycles only, to preserve as much energy as possible. Amongst these scavenging techniques, harnessing energy from vibration is probably the most efficient approach.

### 4.2. Internet of Things Systems

Sensor nodes described in the previous section, typically send their data to a sink node, which in turn sends it to some sort of processing centre. These two components (sensing and processing) are not explicitly unified, from a WSN perspective. Some unified solutions include:

**Thingspeak** [35]: Thingspeak is a platform which enables users to upload information from their sensor nodes to the Cloud. Users create an account on the platform, register their devices, and get access to their sensor data as desired. The system allows querying by location, allowing the user to have access to data from various locations in the world.

**iOBridge** [36]: In contrast to Thingspeak and Cosm, where libraries for different sensing platforms are available, iOBridge develops its own hardware modules that are connected to the Cloud, which in turn can be accessed through web interfaces, for remote monitoring and API's allow integration of the collected information into other web services or third-party applications, for example mobile applications for controlling these ioBridge devices remotely from smartphones such as Android and iPhone.

In contrast to the systems described above, there are also proprietary solutions. These include HPCense [37] and Smarter Planet [38]. HPCense's ultimate goal is to deploy more than a trillion sensors around the planet to collect a range of information such as seismic activity. Smarter Planet is an IBM initiative which encourages companies to adhere to their solutions to improve the efficiency of their current systems. These include building management solutions and IT infrastructures for scalable data storage.

### 4.3. Cloud of Things

The Cloud is a vital component of smart cities. As sensors collect terabytes of information, this data needs to be aggregated and processed. With the recent technological advances in mobile computing, in the form of smartphones, the Cloud has become a critical element for data storage and processing. In simple terms, the Cloud is a collection of platforms and infrastructures on which data is stored and processed, allowing users to retrieve and upload their data for a specific mobile application or computer program, at any location with available Internet access. Hence, the Cloud is a pool of resources accessible via the Internet.

The ability of outsourcing processing power is particularly appealing, because resource intensive applications can be dispatched to the Cloud instead. From the perspective of ubiquitous computing and sensing, the Cloud is essential.

For smart cities, combining the Cloud and sensors is crucial, so that the sensing data can be stored or processed. In [39], a high-level architecture to interface sensors and the Cloud is described. The exchange of data follows the Sensor Web Enablement (SWE) standard.

In the context of Cloud computing, all these platforms can be classified as either infrastructure as a service (IaaS), platform as a service (PaaS) or software as a service (SaaS). Infrastructure as a service (IaaS) consists of providing storage or processing capabilities through the use of virtual machines; platform as a service (PaaS) comprises a platform with execution environment and databases; and software as a service (SaaS) provides facilities for users to access their applications or personal files on the cloud. The integration of sensor devices into the Internet requires IP compatible protocol stacks.

**Figure 3.**
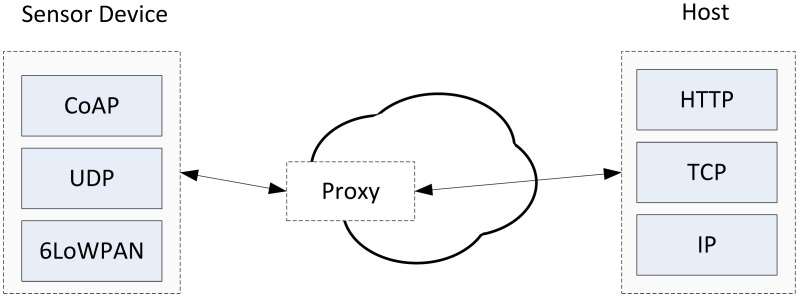
CoAP to HTTP conversion through a proxy in the Cloud.

The Constrained Application Protocol (CoAP) is an application layer protocol, specifically for energy constrained devices, described in [40]. The device in question (in this context, a sensor node) communicates with a CoAP client, which in turn communicates with a web server. CoAP and 6LoWPAN allow sensor nodes to be integrated into the web, through the use of proxies for HTTP to CoAP conversion. In this context, if sensor nodes running CoAP are connected to the network, sensing data could be retrieved by a web browser through *GET* requests, in the same way a HTTP agent is queried. Figure 3 illustrates how CoAP and HTTP co-exist.

Efforts are made to integrate these services into each layer of the protocol stack, such as in Sensinode [13], where these are combined in the protocol stack. IoT standardizing efforts are also a challenge as a large number of companies are involved with IoT development.

Other standardizing efforts include the Sensor Web Enablement (SWE) framework [41], by the Open Geospatial Consortium (OGC), with the goal of seamlessly integrating sensor nodes independent of hardware platform, and defining structures for describing measured data from sensors to a suite of web services available to the platform.

When using smartphones as ubiquitous sensing tools, standards have to put in place for efficient data exchange between devices. In [42] a performance analysis on Sensor Web Enablement (SWE) protocols for data exchange between mobile devices for environmental monitoring applications is presented. SWE protocols are based on XML, which introduce a considerable amount of overhead. By analyzing the execution speed of the XML parsing process, and comparing to alternative formats, such as JSON, or EXI, with reductions in the file size in the order of 40% to 90%, as well as faster parsing times, it was concluded that EXI-C, even though it adds considerable overhead in processing times, results in greatly reduced message lengths. Apart from the integration of sensor devices onto the Web, another issue is the large volume of data due the large number of sensors.

An example of Cloud architecture to solve the high volume data issue, named CLEVER, is presented in [43]. This architecture allows the virtualization of different types of sensing structures so that a common interface can be used for multiple sensor networks. It provides services for sensing, storage and computation. From a cloud perspective, CLEVER is mainly an IaaS solution. A module named C-SENSOR is responsible for managing the sensors' physical resources and abstraction/virtualization. C-SENSOR is compliant with SWE and it interacts with sensor data in an event-based or on-demand fashion. A Host Manager directly interfaces with the sensor network and a Cluster Manager, which interacts with the system's front-end and database. Each sensor network is virtualized as a virtual pervasive element (VPE), which is an integral part of the Host manager.

## 5. Applications

### 5.1. Water Distribution Systems

Water (along with electricity) is one of the most important resources in urban areas. From supplying residential customers to public infrastructures, such as public parks, the distribution of water has to be done efficiently and has to be regulated. Furthermore, efficient quality control has to be performed to ensure that water is safe for human consumption. A typical water distribution system consists of a water collecting point (such as a river or lake), storage facilities (such as reservoirs) and a distribution network, which typically consists of pipes (placed either above or underground and underwater) connecting water collecting points, storage facilities, to the customers' premises.

**Figure 4.**
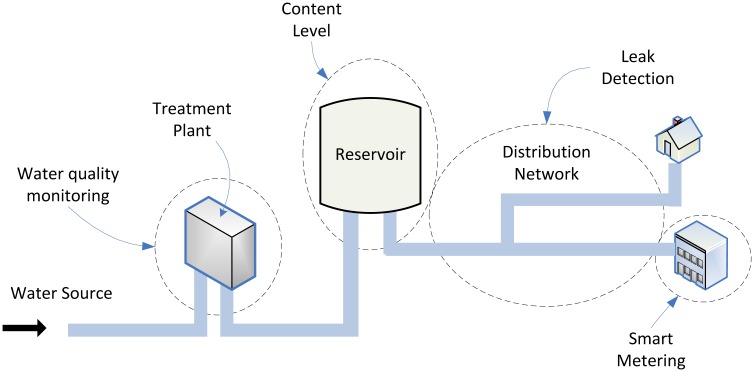
Applications of sensing in water distribution systems.

These distribution systems are in essence non-intelligent. For instance, if there is a leak in one of the distribution pipes, it can be tricky to diagnose the system early enough to detect the fault, especially if it is not readily visible (in case of underground pipes for instance). Advanced sensing enables a more reliable fault detection system. Possible locations to deploy sensors and parameters of interest, from a monitoring perspective, in water distribution systems, are shown in Figure 4. Examples of such applications include monitoring the content level in reservoir tanks, leak detection and monitoring the water quality at specific points along the distribution system.

For pipeline monitoring, the method shown in [44] consists of sensors deployed around the pipeline for continuous monitoring. Typically, three parameters used to indicate faults in pipelines are vibration, pressure, sound (made by the liquid as it leaks) and water flow [45]. By monitoring these parameters, a leakage can be successfully detected. In [46], a WSN monitors hydraulic, flow and acoustic data, as well as water quality, with nodes deployed at strategic locations along the pipeline and also in sewers to determine content levels.

**Figure 5.**
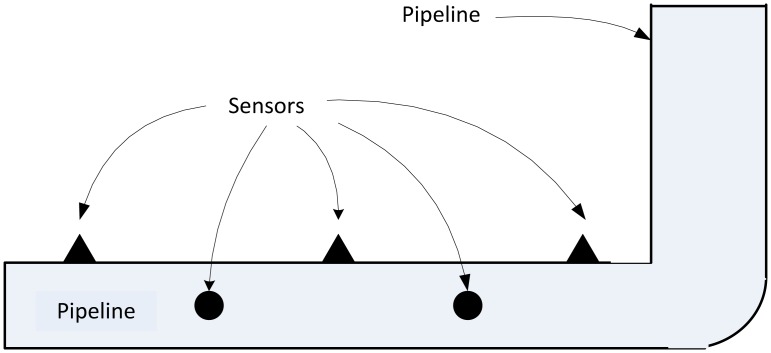
Vibration, pressure, and flow sensors in a pipeline. These are used to monitor the integrity of the pipeline, as described above.

For pressure sensing, this system uses a piezo-resistive sensor and for measuring water pH (for quality monitoring purposes), a glass electrode is used. For water level monitoring, an ultrasonic sensor was placed on top of the collector, and two pressure transducers were placed at the bottom [45]. Additionally, using dual-axis accelerometers, vibration data is collected. These sensors are deployed along the pipes as shown in Figure 5.

The collected data is then analyzed to search for leaks. By analyzing the pressure data using Haar Wavelet transforms, pressure pulses along the pipe can be detected to determine whether bursts occurred and their approximate location. High magnitude noise in the acoustic signal is also an indication of a leak. Since sensors are typically placed apart and by cross-correlating the data collected by neighbor nodes, and considering time differences due to the sensors' spatial positioning, the location of a leak can be determined. As these analysis methods require considerable processing resources, the data collected is analyzed remotely and not locally on the sensor nodes. A device to mitigate effects from the leak can be activated when an anomaly is detected. In the context of pipeline monitoring, this could consist of a device that would instruct an electro-mechanical device (*i.e.*, actuator) to act in order to restrict the flow of water to sections of the pipe which could have been compromised by the leak. Other methods consist of placing meters inside the pipe to determine liquid flow [47].

Hence, integrated sensing, processing and actuators results in an intelligent structure, where the actuator's decisions do not require human intervention. The data is collected by the sensing agent, analyzed, classified and an intelligent decision is carried out by the actuator.

### 5.2. Electricity Distribution Systems

Conventional electricity distribution systems are in essence non-intelligent. Typically, these energy distribution infrastructures provide unidirectional flows, from the generating station to the customers. The amount of supplied electricity is estimated or predicted, through previous available data, in order to perform load shedding for instances where the demand exceeds the supply.

An improvement to the conventional distribution scheme is a bi-directional system, in the sense that electricity flows from the utility to the client's premises and *vice versa*. A more efficient use of energy is then possible, whereby electricity can flow back to the utility (to be stored for later use) in cases of low demand. These systems are collectively known as smart grids. The concept of distributed generation enables a scenario where, instead of having a centralized generation scheme, additional electricity generation points can be located closer to customers, therefore reducing the impact of losses on electricity transportation over long distances. Additionally, this enables more innovative demand response programs to be put into action.

**Figure 6.**
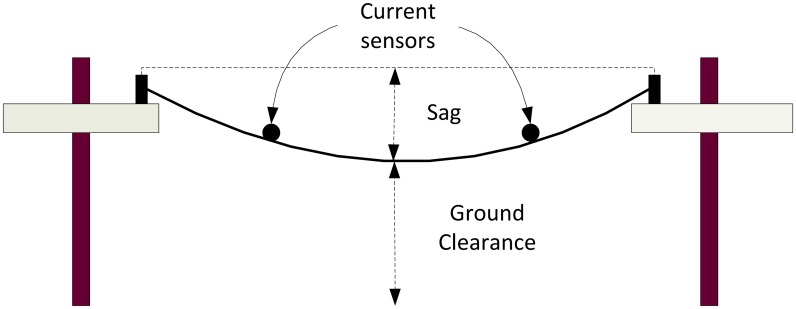
Location of current sensors in a transmission line to monitor the line's state.

In contrast to conventional power grids, smart grids introduce many improvements. Firstly, smart grids have self-monitoring capabilities [48], where sensors can be deployed anywhere along the distribution network, in locations such as substations or overhead transmission lines, for instance. Sensor data can then be used to report relevant events (in terms of performance and reliability) back to appropriate entities. An important sensing application in smart grid, amongst others, is the monitoring of overhead transmission lines. By monitoring the lines, utilities ensure that power delivery occurs within safe limits. For instance, systems like STAMP [49] sense the temperature, sag and tension values of an overhead line in real time and determine the state, or health, of the line from these measurements (illustrated in Figure 6). STAMP achieves this using a distributed temperature system (DTS) and thermocouples for temperature measurements, a laser range finder to detect sag and ground clearance, and two load cells to monitor tension. These parameters have to be carefully monitored to ensure that the conductor is operating at its ampacity rating, which is dependent on temperature.

Furthermore, parameters such as voltage, current and phase can be determined through voltage and current sensors, such as medium voltage (MV) sensors [50], placed directly on the transmission lines, or non-intrusive sensors, which measure magnetic field variations. An example of a stick-on, low-cost sensor for such purposes is proposed in [51], which can be readily deployed in transformers and overhead conductors.

### 5.3. Smart Buildings and Homes

In an effort to promote green living and sustainability, there are various initiatives to decrease energy consumption in buildings. In this context, methods to significantly reduce energy consumption in buildings are various, from intelligent systems to smart design. These are necessary because buildings (especially with the easy accessibility to inexpensive electrical appliances or other electronic devices such as computers, *etc.*) consume a very large portion of generated electricity. In particular, heat, ventilation and air conditioning (HVAC) constitutes the largest contributors to increased consumption. Hence, sensing is crucial in buildings for appropriate actions to reduce consumption can take place. For building automation systems, for instance, before an appropriate action can take place (which could be dimming lights or switching of air conditioning) the state of the environment has to be properly assessed. At the heart of household energy sensing are smart meters. Energy sensing measurements are interfaced with a smart meter for accurate consumption reports. Sensing can also aid in demand prediction.

#### 5.3.1. Approach to Energy Sensing

Accurate power usage measurement at the load side requires appropriate sensing. Additionally, the existence of a bi-directional grid enables the use of smart meters at customer premises. Determining how much power electrical appliances and electronic devices are utilizing is crucial. To this end, sensors can be placed on these electrical appliances for accurate measurements.

Three different approaches for energy sensing at the customer's premises are: distributed direct sensing, single point sensing and intermediate sensing [52]. With distributed sensing, a sensor is placed on each appliance, which means that although measurements will be more accurate, it is an expensive approach due to installation and maintenance costs. Single-point sensing, in contrast to distributed sensing, measures voltage and current entering a household. Although not as accurate as distributed sensing, it lowers costs significantly. By monitoring raw current and voltage waveforms, and subsequently performing feature extraction on these measurements, a classification algorithm can be used to determine which appliances are on or off, by comparing the measurements to existing device signatures. Intermediate sensing falls between direct and single-point sensing. For intermediate sensing, smart breaker devices are installed in a household's circuit panel to provide a more detailed analysis of consumption. Other sensing approaches described in [52] are based on voltage signatures, where voltage noise signatures or current signatures are used to classify operation of electrical appliances, by observing the spectral envelope of the harmonics and comparing these observations to existing templates.

#### 5.3.2. Correlation between Demand Prediction and Weather Conditions

The concept of Smart World as an interconnection of smart infrastructures is introduced in [53]. This concept includes sensing a range of parameters describing the earth's state, such as environmental pressure, temperature, radiation and similar parameters which can be exploited by the global community to improve the efficiency of services provided to the community.

For instance, accurate demand prediction is necessary to avoid rolling blackouts or similar measures when demand exceeds supply. With regard to demand prediction, the correlation between weather (which is typically characterized by temperature, pressure, humidity and wind speed and direction) and demand, or human behavior, is of particular interest. In [53], the effect of weather on electricity consumption is investigated. It is found that the highest correlation weather variables with power consumption are temperature, global solar radiation and humidity [53], for different times of the day and seasons. The work in [53] specifically focuses on smaller areas, such as micro-grids. This is correlated with the usage of appliances, as heaters, geysers and air conditioners tend to comprise a large percentage of a house's electricity consumption, and the use of these appliances is correlated with seasons. By using environmental data and intelligent agents to help predict load, it is possible to achieve greatly improved demand prediction.

#### 5.3.3. Occupancy and User Context

The most basic action when it comes to occupancy is to determine whether there are people in a room or not. Typically, this can be achieved through a combination of sensors, as shown in [54]. These sensors include passive-infrared (PIR) sensors [54] or more sophisticated camera based methods such as the methods shown on [55], where people can be actively counted to determine occupancy numbers and locations. PIR detectors are used in home alarm systems for motion detection (*i.e.*, to detect whether there is a person in a room). Apart from occupancy, another important feature is the user context, as highlighted in [56]. This can be achieved by behavior activity classification using video cameras for instance, as shown in [57]. The data from these sources enables an appropriate plan of action with regards to the people in the room. For instance, lights in corridors, elevators, and or other facilities can be dimmed if there is no human activity, hence reducing the overall energy consumption.

#### 5.3.4. Indoor Monitoring

Of interest are also the buildings' ambient temperature and ambient lighting. For instance, with light emitting diodes (LEDs) lamps, the intensity can be reduced in cases where ambient light, from open windows for instance, is sufficient. These windows are made of tintable material which reacts to changes in the environment [58], and can reflect or absorb light, depending on an external electrical stimulus. This is a simple example of how energy consumption can be reduced by smart design.

Indoor temperature can also be monitored so that parameters in the HVAC system can be adjusted to meet specified temperatures, described by predefined set-points. For these purposes, humidity and temperature are deployed in strategic positions to collect the required data for actuation.

The smart thermostat proposed in [59] is an example of a combination of sensors and actuators in the smart building context. The thermostat is coupled with motion sensors to detect occupancy and then creates an optimal setback schedule based on this collected data for autonomous operation.

In [60] a ZigBee based HVAC control system is described for ensuring an adequate comfort level in buildings, using machine learning techniques. Sensor nodes are equipped with temperature and humidity sensors, from which data is collected at one-minute intervals and sent to a logging system. This data is clustered and matched to user feedback, where users can indicate their current comfort level. In the control phase, the control system and clusters classified as uncomfortable can be moved to the closest comfortable cluster.

Apart from monitoring and controlling HVAC systems, it is also necessary to monitor electrical appliances, to detect any faults and to be able to assess current operating status remotely, and having the appliances wirelessly connected to each other in a home area network (HAN). Furthermore, these systems can be connected to social networks to share notifications, enabling remote monitoring. An example of such a system is described in [61]. All appliances have a set of sensors (to measure humidity, temperature or pressure) mounted on them, which are connected to a local sensor host (LSH), and are equipped with 802.3 u and 802.11 g interfaces, so that these nodes can be connected to an available wired or wireless network, which is connected to the Internet. Each appliance is fitted with a specific set of sensor types. For instance, a refrigerator can be fitted with pressure and acoustic sensors on each tray to determine the quantity of food. Examples of notifications from appliances include warnings from washing machines upon completion of a washing cycle.

#### 5.3.5. Smart Building Evacuation

Another aspect of smart buildings is smart evacuation systems. Conventional evacuation systems activate an alarm when an alert for an emergency situation is triggered. This model assumes that people within the building are aware of the routes to exit the building. A more efficient way to perform building evacuation is to count the number of people and direct them through the quickest routes by considering congestion information, obtainable from a variety of sensors, such as beam sensors or video cameras. Examples of such WSNs are described in [62] and [63], using beam sensors and detection of tags worn by the people in the building, respectively. Although not as accurate, these methods are preferable to video camera based methods, from a processing cost perspective. Instead of using these systems stand-alone, they can be interfaced to the building's infrastructure, to obtain an estimate of the number of people through existing occupancy and user context functionality, typically based on motion sensors or video cameras, if available.

### 5.4. Monitoring Bridges and Seismic Activity

All public infrastructures have to be monitored to assess their condition. Even if these infrastructures are monitored by personnel through scheduled visits, a visual inspection is often not sufficient, as anomalies may still go undetected. A more efficient approach to monitoring is to install a variety of sensors across the structure to continuously monitor its state and determine its health.

There are a variety of parameters which characterize a structure's state. In [64], the most common parameters for monitoring of structures are listed as chemical (pH, oxidation, and corrosion), mechanical (strain, stress and deformation) and physical (temperature and humidity). A variety of sensors capable of measuring these parameters can be deployed in large numbers across a structure for accurate monitoring, but these can incur a significantly high cost. Recently, fiber optic sensing (FOS) has received substantial attention from the research community.

Recent approaches for SHM include using sophisticated fiber optic sensors [65]. In bridges, for damage detection, optic fiber sensors can be used for structure monitoring. An optic fiber sensor works by detecting the change in the detected signal from a transmitted signal, after it has passed through the structure of interest, and is able to measure strain, pressure and temperature, by interpreting changes in wavelength. This typically requires the installation of fiber into the structure to be monitored. These distributed sensors work on the basis of back-scattering. As light propagates through the fiber, some of it is scattered back along the length of the fiber. Since the properties of the back-scattered light depend on the strain, deformation or temperature of the fiber, by using this information one can determine the state of the structure where the fiber is installed. A fiber distributed sensor is equivalent to having a large number of sensors deployed along a line with high resolution, since all points along the fiber can be considered sensing points, as described in [66]. Other approaches to sensing for SHM use WSNs with sensor nodes equipped with multiple accelerometers to sense vibration along a 1,300 m long bridge [67]. The appeal of these systems is the relatively low cost and ease of installation in comparison to more sophisticated sensing solutions. These sensors were deployed over the main span and tower, and collected information of structural accelerations from wind load. Additional approaches include the use of thermal responses and combination of multiple sensors such as strain gauges, strain-gage displacement and force sensors, as well as non-intrusive sensing methods using video cameras [68]. These computer vision based systems can be used for instance for structural crack detection [69].

Another important application of sensor networks in smart cities is monitoring the ground to detect any anomalies. These allow early detection of earthquakes or landslides, which can be used for early warnings in emergency situations. In [70] a wireless sensor network is suggested to monitor sub-soil activity. Sensor nodes are buried underground. In contrast to other monitoring techniques, which rely on sensors attached to the sensor node, the technique proposed in [70] relies on how the propagation signal is affected by soil conditions. By considering changes in the received signal strength (RSS), it is possible to infer events based on how each soil parameter affects the signal. In particular, water intrusion, detection of relative motion and relative density change can be detected using this technique.

### 5.5. Environmental Monitoring

To ensure quality of life, as well as safety, environmental monitoring is of utmost importance. By monitoring the quality of air, quality of water, and other parameters such as humidity, temperature and ambient carbon dioxide level, as well as other harmful gases, it is possible to assess anomalies in the environment; therefore ensuring pollution is kept to an acceptable level. In particular, reducing the level of carbon emissions, which is a serious threat to our planet is one of the main goals of smart cities. Environmental monitoring requires deploying a variety of sensors outdoors, in locations such as parks and rivers, *etc.* With the proliferation of smartphones, it is possible to use them for collaborative environment monitoring. Furthermore, it is necessary to better understand how human behavior is influenced by weather conditions, with regards to mobility and other aspects.

In [71], the relationship between human behavior and weather is studied, in an attempt to gain a better understanding of the dynamics of an urban system. Data characterizing air quality and weather was collected from environmental monitoring stations and analyzed in conjunction with network mobile traffic data generated during a period of 20 days. It was demonstrated that by combining data from different ubiquitous sensing sources (environmental data and mobile traffic data), it is possible to analyze environmental and social dynamics, which can be used to improve on a lot of aspects in the context of smart cities, such as public transportation and other services.

### 5.6. Intelligent Transportation Systems

With the steep increase in car numbers over recent years, there is an increasing need for efficient traffic management, to avoid traffic jams and optimize traffic flow, especially at intersections. A conventional way to regulate traffic flow is through the use of traffic lights. These typically have fixed switch interval times (*i.e.*, from red to green and to yellow), which are not adjustable to traffic conditions. Traffic jams have significant impacts on fuel consumption due to the frequent starts and stops, as well as increased carbon emissions. An adaptive scheme, dependent on traffic conditions, is more attractive. In this context, a method to estimate the number of cars approaching an intersection could generate information for switch interval times to be dynamically adjusted based on traffic conditions. For such an intelligent system to be realized, efficient methods to detect traffic (*i.e.*, count the number of cars) are required. Approaches where traffic lights and stop signs are completely removed have also been proposed, as described in [72]. Such an approach is based on an intelligent intersection where vehicles, which are wirelessly connected to each other, can communicate with each other and use decision-making for collision avoidance. Using a time-slot for each vehicle, crossing of intersections is coordinated amongst vehicles [73]. A vehicle which misses its time-slot has to stop when it reaches the intersection and wait for a new time-slot. The wireless communication can be performed through RFID, as proposed in [74]. In [74] a system to control a vehicle's speed autonomously based on the traffic signs is described. RFID was used for communication between the vehicle and traffic signs, with RFID tags placed on traffic signs and RFID readers placed on the side of the doors of the car, and a Hall Effect sensor placed on the vehicle's wheels for speed control.

**Figure 7.**
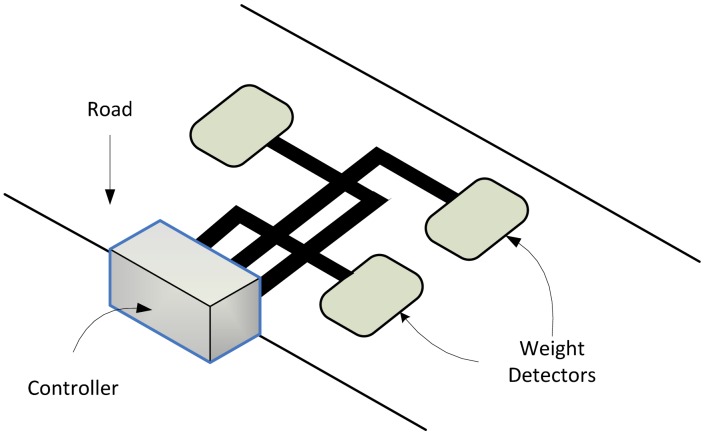
Weight in motion system. Weight information recorded by the weight detectors is sent to the controller via wired connectors, such as a fiber optic cable, for storage and processing.

Inexpensive methods of sensing cars involve induction loop detectors which are buried in roads. Loops detectors can be used to detect the presence of metals. When a vehicle approaches the loop, the loop's resonant frequency increases, and this change in frequency is interpreted as a vehicle. The increase in frequency differs according to car size (or height), enabling an estimate of the kind of car which has been sensed. This information can then be used to estimate vehicle speed or other parameters, which can be useful for traffic flow management. To estimate a vehicle's weight, other methods such as weight in motion (WIM) can be used. A WIM system captures vehicle gross and axle weights using sensor mats on road surfaces. Various types of WIM systems are discussed in [75]: piezoelectric systems (which detect changes in voltage due to pressure); capacitive mats (two inductive loops and one capacitive weight sensor); bending plates (which record the strain on the plate due to the dynamic load of the vehicle); load cells (which use a single load cell with two scales for each side of the vehicle's axle); and optical WIM (where pressure on the optical fiber creates a phase shift in polarization modes which is directly related to the load on the fiber). All methods highlighted above usually carry significant installation and maintenance costs. A WIM system is illustrated in Figure 7.

Less intrusive (but not as accurate) and more sophisticated methods involve video cameras. These approaches are more attractive mainly because cameras can be easily installed and the level of required maintenance is low, compared to previously described approaches. Furthermore, in instances where there are surveillance cameras installed, these can be used for intelligent transportation applications. A wireless real-time traffic monitoring system is proposed in [76]. The system consists of multiple standard CCTV cameras and personal computers (PCs), which process the captured data from the cameras. Each camera is controlled by the PCs, and each PC implements all the necessary algorithms for recognition and tracking.

The system was tested in different environments, such as airports and tunnels. One of the drawbacks of computer vision-based methods is their performance dependency on environment conditions, such as lighting and occlusions. Dealing with occlusions or weather conditions is challenging in computer vision, so such scenarios have to be handled with care. For instance, in the presence of fog or rain, vehicle detection or counting is more challenging. For night-time scenarios, street lamps and vehicle lights also have to be considered [77]. In particular, street lamps, traffic lights can be used for vehicle sensing, as vehicles can be detected by considering the distance between vehicle's headlights.

### 5.7. Surveillance

With the availably of conventional CCTV systems for surveillance purposes, an infrastructure for smart surveillance systems already exists. Security, especially in urban areas, is of paramount importance. Although these cameras are typically connected to a digital video recorder (DVR), most only record and do not have any built-in intelligent processing capabilities. Furthermore, these systems are run by human operators who are prone to loss of concentration. The benefits of using smart surveillance are obvious. For instance, by monitoring peoples' actions, it is possible to determine whether violent actions are taking place, and even trying to identify and recognize the people involved.

In this context, smart surveillance systems could alert users by triggering alerts when events of interest take place. Additionally, monitoring people's behavior can help in determining pedestrian traffic patterns which can be of help in the design of future pedestrian facilities or modification of current facilities.

In the instance of gatherings, it is important to have crowd control schemes in place, especially in cases where there are large numbers of people, such as in concerts, or other public areas such as airports. This is especially relevant in emergency management systems or pedestrian crowd monitoring, especially at night-time. To track and detect pedestrians at night, an infrared video can be used [78], as infrared cameras work on the basis of temperature and not on reflected light, hence infrared based computer vision techniques are less susceptible to lighting.

Another important aspect of crowd surveillance, especially in security applications, is to determine whether people are carrying any objects, such as suitcases, *etc.* This allows determining whether a person, for instance, is entering a restricted area while carrying an object which they are not allowed carrying in specific facilities. A framework to detect objects carried by people from video sequences is presented in [79]. This method works by considering irregularities in a person's silhouette. This is performed by comparing a template of an individual walking in the same direction as the individual being observed, with any protrusions noticed considered as possible pixels for carrying objects.

Another important aspect of crowd control is determining violent behavior. Monitoring of fights, riots and similar events is important for safety reasons. A crowd behavior classification algorithm is proposed in [80].

A semantic video surveillance system able to autonomously detect abnormal situations, able to run on low resolution (320 × 240) cameras is described in [81]. The system's cameras run motion detection algorithms with are used to extract information from the video and format it in XML, aggregating several frames, which are then used to perform route detection, which consists of extracting information of objects which enter or exit the scene. Finally, a semantic reasoning module is used to classify the semantic data obtained from semantic translation to determine whether there are anomalies such as cars driving on the sidewalks or similar situations which could indicate abnormal situations. This system can be used to detect pedestrians crossing streets without using crosswalks or walking on railways and vehicles going in the wrong direction. Hence, this system can be deployed in different scenarios only changing its rules' set to fit that specific environment.

### 5.8. Public Services—Law Enforcement and Fire Fighting

The conventional law enforcement model is impractical and inefficient. Investigations and other processes within law enforcement are aggravated by human incompetency and lack of automation in processes. For law enforcement, IBM has solutions within the Smarter Planet initiative, integrating investigations, geographic information systems (GIS) and intelligence analysis [82], enabling information sharing across agencies, resulting in a more efficient and more coordinated law enforcement scheme. Another public service that can be improved by use of technology is fire-fighting. Fire fighters risk their lives in the line of duty, due to the harsh conditions in which they have to carry out their work.

Conventional fire-fighting can also be improved, as shown in [83]. This system consists of a TelosB based wireless sensor network, where the nodes are placed on firefighters' bodies. Each TelosB mote is equipped with sensors to measure temperature, carbon dioxide, and hydrogen and hydrocarbon concentration. Using RSSI based localization, firefighters are aware of their position relative to other fighters within the network. This also enables computation of escape paths, so that firefighters can exit the work location safely.

### 5.9. Health Care

One of the most important applications of sensor networks is in healthcare. To facilitate management in hospitals or clinics, sensors can be used in these environments for various applications. In particular, wireless body area networks (WBANs), is a very active research field. Body area networks consist of inter-connected sensors placed in different parts of the human body. Hence, with sensors attached to one's body, either internally or externally, it is possible to monitor an individual's vital signs remotely. Examples of parameters which can be monitored include electrocardiography (ECG), respiration, skin conductance and skin temperature [84]. The advantage of WBANs is their capability to upload this data to remote logging systems so that the collected data can be viewed by a medical practitioner in real-time. Individuals with heart problems, for instance, can be constantly monitored and alerted if there are signs of an anomaly. Activity monitoring and classification, which is useful in health monitoring, can also be performed by WBANs. But healthcare applications using sensing technology are not limited to WBANs.

Other examples of sensing applications in healthcare involve emergency response [85]. In [85], a protocol for emergency reporting using cognitive wireless sensor networks is proposed. This protocol is modeled after ants' behavior and is used to coordinate cluster-heads actions more efficiently, which results in a decrease in reporting time delays.

A framework for long-term activity monitoring is proposed in [86]. The system is able to recognize user activity in real time and classifying activities, using an array of sensors deployed in the home environment and attached to doors, refrigerators and cupboards, as well as light sensors. From activity classification, it is possible to infer whether the person has a certain disease or not. For instance, if frequent sleep is detected, it is possibly due to fatigue, which could indicate that an individual has diabetes, for instance. This framework can be used for disease prediction based on day-to-day activities.

A user-context recognition system is presented in [87]. It uses Hidden Markov Models (HMM) to assess users' stress levels. Users insert their stress levels into a questionnaire through a smartphone application. The entered data is matched to smartphone's context data, such as location and activity information, which is then used for HMM training. Stress levels can then be classified after the training phase.

### 5.10. Crowd Sourcing

Crowd sourcing consists of outsourcing tasks to a group of people, or crowd, in an attempt to collaboratively completing tasks quickly. The smart city not only consists of smart infrastructure and sustainability: smart people are also an integral part of the smart city. With the recent proliferation of social platforms and mobile computing, crowd sourcing becomes a viable medium for exchange of skills and services. Workflows, in crowd sourcing, consist of tasks which have been unified. For crowd sourcing to be effectively carried out, workflow processes have to be designed so that jobs can be broken into tasks and efficiently dispatched to human actors, and these workflow processes have to be ideally integrated into social platforms. To this end, various tools have been developed. In [88], a collaborative workflow tool for Amazon Mechanical Turk is described. It allows requesters to view tasks' current status and reduces required planning by requesters. CrowdScape [89] attempts to include user behavior into the crowd sourcing process, in an attempt to improve work efficiency. User behavior whilst completing a task is considered in conjunction with worker outputs, using machine learning. Actions considered to characterize user's behavior include frequency of mouse click events, key presses and scrolls. By correlating the user behavior with good work outputs, it is possible to identify traits of good workers, helping make crowd sourcing task assignments more efficient.

With smartphones becoming increasingly more powerful in terms of resources, crowd sourcing using smartphones phones has been thoroughly investigated. For instance, as users regularly update their location status on social networks like Twitter and Facebook, based on this location information, it is possible to aggregate this data, enabling tasks to be dispatched to people in specific locations. Furthermore, by using sensor data on a smartphone, it is possible to determine a person's state (*i.e.*, whether the user is stationary or not), as well as environment. This data can be collaboratively used to obtain information regarding the weather with location information [90].

Given the number of smartphones users in the world, this enables pervasive sensing. With smartphones fitted with a variety of sensors such as GPS, gyroscopes, accelerometers and compasses, smartphones enable a variety of crowd sourcing applications, which will be eventually augmented by the Internet of Things. In particular, collaborative data collection is a popular crowd sourcing application.

Examples include MobSens [91], which combines four applications: PollutionSpy to monitor air pollution; NoiseSpy which captures noise levels; MobAsthma, a personalized asthma monitoring application; and Fresh, an application which provides a platform to let users discuss issues regarding their environment.

Although crowd sourcing is typically outsourcing work tasks to people, crowd sourcing can also be used for sensing, as described in [92]. Crowd reporting, as described in [92], consists of collecting information from sources such as mobile devices and social networks. The data reported can be in the form of messages which are aggregated and converted into a uniform format, for processing. The data is collected from Twitter as using a set of keywords to select relevant information. This data is then pre-processed, filtered and clustered so that event correlation can be determined.

Although sensing through crowd sourcing (*i.e.*, pervasive sensing) is appealing to a number of promising applications, there are still a number of challenges, which are not necessarily of a technical nature. Privacy, in particular, is possibly the biggest challenge. Additionally, involving the not-so-evident risks related to large-scale data collection as well as legal issues such as data ownership, user protection [93] and trust needs to be insured as entities responsible for analyzing the collected data, can abuse the collected information or use it to harm the user.

### 5.11. Summary of Benefits of Smart Sensing

From the content presented in this section, it is evident that advanced sensing applications in cities enable improvement in process efficiency. Based on this information, Table 3 summarizes some of the benefits of using advanced sensing in smart cities discussed in this section.

## 6. Smart City Pilot Projects

The concept of a smart city as a system that is highly intelligent and autonomous is far from being realized. Even with recent breakthroughs in technology, available technology is still not sufficiently mature for smart cities to be truly autonomous. Nevertheless, there are currently pilot projects of smart cities in development worth mentioning.

Development for Songdo [94], a fully ubiquitous 1,500 acre city in South Korea, started in 2001 and is predicted to be complete by 2015. Smart systems in every building are used to monitor the water and electricity, which also allow residents to connect remotely using their smartphones. Sensing technologies include RFID tags on vehicles, which send signals to sensors on the road to monitor traffic flow, surveillance systems as well as smart street lights, which can be adjusted to pedestrian traffic. Some of the innovative technologies in Songdo [95] include home and building automation where users will be able to control the systems in their houses remotely. Ubiquitous sensors are deployed virtually everywhere, from buildings, (for safety purposes, for fire, *etc.*) to flow sensors, which control the canal in Songdo's Central Park, and on street lights to adjust the level of lighting according to the pedestrians' flow. Smart power in Songdo is generated by natural gas and distributed through a smart grid. Tele-presence facilities are being installed in homes, hospitals and shopping centers.

PlanIT Valley [96] in Northern Portugal is another pilot project for a fully ubiquitous city built from the ground up, which will accommodate 225,000 residents once it is completed. Completion is scheduled for 2015. Ultimately, PlanIT has a goal of having 100,000,000 sensors deployed.

Fujisawa Sustainable Smart Town (SST) [97] in Japan is also another project which is currently under development. Fujisawa is scheduled to be completed by 2014 and fully inhabited by 2018, and development has started in September 2012. It is being developed on 19 ha of land, with the goal of accommodating 1,000 residents. Targets of 70% reduction of carbon emission are planned.

Amsterdam, unlike the previously mentioned fully ubiquitous cities which are being built from scratch, has seen some recent developments en route to make it a smart city. The main driver of this development is Amsterdam Smart City project [98], which started in 2009, with the main goal of increasing green growth using technology. Currently, there are various initiatives within the scope of Amsterdam Smart City; these services are tested in specific city locations, such as IJburb. It includes innovations such as West Orange (a residential energy management system, which is able to let users know about their energy consumption on a per appliance basis) and a smart grid initiative in Nieuw West which services about 10,000 households. In Groningen, there is a pilot project aiming to develop a system which will use GPS data for real time information of bus locations, readily available to users' mobile phones through a downloadable application [99].

The city council of Norfolk has also pilot projects which aim to make data services delivery to citizens more efficient, through the improvement of their data collection and data analysis, to ultimately gain improved customer insight [100]. This project also aims to develop schemes of delivering data to groups of users through localized and personalized web services, based on users' geographical location.

Another pilot project which has recently received significant attention is in Santander [101]. This is collaboration between academia and industry players which aims to conduct IoT research in available large scale test-beds. Currently, various sensor nodes are deployed in the city. A backbone provides the architecture necessary for all communication between these sensor nodes.

Examples of applications enabled by sensor networks in Smart Santander include traffic management, irrigation optimization for parks and gardens, augmented reality and waste management. Eventually, it is envisaged that 12,000 sensors and actuators will be deployed throughout the city by 2013. Currently, there are approximately 2,000 nodes deployed. Examples of implemented trial applications include outdoor parking area management, real time monitoring of light, temperature and environmental noise, as well as participatory sensing using sensors in mobile phones.

In Barcelona, there are also important initiatives [102]. As part of this initiative, for instance, sensors have been deployed in garbage bins. Firstly, these allow remote monitoring of the content of bins, which enable the development of an improved garbage collection system, therefore optimizing garbage collection services. Additionally, built-in sensors monitor the content type in bins and smart humidity sensors enable an automated intelligent water use for public park irrigation. Panels strategically placed announce free parking places, saving residents' time in locating parking spots. Sensors on streetlights detect presence and adjust the light intensity accordingly as well.

In Malaga, another smart city initiative in Spain, smart city initiatives aim to achieve a reduction of more than 6,000 tons of carbon dioxide annually [103]. To this end, Malaga has invested significantly in the city infrastructure, including investments in a fiber optic network, municipal Wi-Fi networks and geographic information systems for the virtualization of urban indicators. The current deployment includes more than 17,000 deployed smart meters.

Vienna is another city with a number of initiatives from the smart city perspective. The main initiatives in Vienna deal primarily with energy efficiency, carbon footprint reduction and climate protection [104]. A large number of projects are currently underway in Vienna. Table 4 summarizes the goals of the cities discussed in this section.

## 7. Challenges

There are various challenges related to sensing in smart cities, both of a technical and social nature. Some of these challenges are:

**Addressing and coordination issues between sensor nodes:** When nodes are deployed in millions, how can they be efficiently managed? Although 6LoWPAN enables IPv6 addressing support on IEEE 802.15.4, efficient coordination (in terms of routing, for instance) between such a high number of devices is a big challenge.

**Security:** Enabling technologies for sensing applications have a number of issues which have to be considered within the context of smart cities. These networks will be prone to cyber-terrorism and cyber-vandalism. Some of these issues are highlighted in [105] and [106].

**Data ownership and privacy:** Who will own the rights for all data collected by these sensing applications? For instance, if the electricity utility has access to an individual's energy consumption data, can that data be used without one's consent?

**Trust:** Can individuals trust the entities responsible for aggregating and storing their data? For instance, the Cloud can be considered a central information system where access to data is typically controlled by the company which owns the infrastructure. Can these companies be trusted?

**Social Issues:** Residents have always been actively involved in a city. For instance, there are collaborative initiatives where people come together and help keeping the city clean by promoting no littering, *etc.* Will smart cities (with all the automated processes and sophisticated technology), create some sort of disconnect between the people and the city? Then there is the issue of education as well. Is the average person educated enough to use and understand all these systems?

**Centralized control:** It is evident from the content of this paper that we are moving towards a centralized control scheme, where all services are being progressively aggregated, to be managed by one huge central system. This translates to complete control by the governing bodies, which can be used to illegally track people or invade someone's privacy. This is related to the trust and privacy issues previously mentioned.

**Costs incurred in upgrading current cities:** How expensive will it be to upgrade current cities to make them smart cities? As smart cities are heavily dependent on communication and other infrastructures, it will certainly be very expensive to update all the current systems.

The reader is referred to [48], where the authors discuss the major technical challenges of WSNs in smart-grid applications.

## 8. Conclusions

In this paper, the role of advanced sensing in smart cities was discussed. An array of applications in smart cities which can benefit from advanced sensing were described. These include infrastructure health monitoring, electricity and water distribution systems, transportation systems and surveillance, amongst others. The state of the art in each of the considered applications is reviewed and inherent challenges are highlighted. In a world where carbon emissions have to be reduced for greener living and sustainability is promoted, there are a still a lot of challenges to be resolved.

It is evident that the evolution of technology will play a major role in advanced sensing, as the evolution of hardware required for sensing applications will certainly be driven by these technological advances. Even though there are pilot projects for smart cities, there are various challenges. These challenges require a holistic approach for solving, which will involve multi-disciplinary collaborations. For instance, standardization efforts should consider all involved parties, such as municipalities, utilities and other services providers so that unified solutions in terms of sensing and communication infrastructures are put in place. It will be inefficient to have separate infrastructures (in terms of sensing platforms), but it will also be challenging to unify these services under a single infrastructure (*i.e.*, have the same network on the water distribution system as well as the electricity system).

Furthermore, non-technical challenges regarding social issues, like education, also need to be considered. In the context of smart city, it is expected that people will have a certain level of skill to understand these systems and be able to use them. For instance, the innovative solution brought about by advances in sensing technologies, such as improved demand prediction and smart metering, result in a more efficient electricity distribution system, where the customer also plays a role, by adhering to incentive programs. But to what extent does the customer want to be really involved? Some individuals might not even take advantage of such services. These factors should be taken into consideration before services are implemented.

Although there are already existing systems and initiatives for smart cities, the infrastructure to implement a fully ubiquitous city will be costly. Furthermore, the cost of restructuring existing cities to fit the fully ubiquitous city model will incur much higher costs.

The first pilot projects mentioned in this paper will serve as a model for fully ubiquitous cities to be implemented in future, and will provide insights into additional challenges in the realization of smart cities. Still, there are numerous challenges to be solved before fully ubiquitous cities become a reality.

## Figures and Tables

**Table 1.** Comparison of communication standards.

	**Dash7**	**ZigBee**	**LTE**	**3G**	**NFC**
**Standard**	ISO/IEC 18007-7	IEEE 802.15.4	3GPP-LTE	Various	ISO/IEC 18092
**Frequency (MHz)**	433	868/915/2400	700-2600	700-2600	13.56
**Penetration**	High	Lower	Low/High	Low/High	High
**Range**	1 km	500 km	Several kms	Several kms	10 cm
**Data Rate**	200 Kbps	250 Kbps	100 Mbps +	3.6–21 Mbps	106–424 Mbps

**Table 2.** Comparison of various sensor motes (specification data sourced from devices' datasheets).

	**I-Mote**	**Mica2**	**Iris**	**Sunspot**	**Waspmote**	**I-Mote 2**
**Processor**	32-bit ARM 12 MHz	ATmega 128 16 MHz	ATmega 128 16 MHz	ARM920T 180 MHz	ATmega 128 16 MHz	ARM 11 400 MHz
**Transceiver**	Bluetooth	Chipcon (CC1000) 868/916 MHz	Atmel AT86RF230 2.4 GHz	Chipcon 2.4 (CC2420) 2.4 GHz	ZigBee/DigiMesh 868/900/2,400 MHz	Chipcon CC2420 2.4 GHz
**Memory** **(SRAM/Flash)**	64 K/512 K	4 K/128 K	8 K/128 K	512 K/4 MB	8 K/128 K	256 K/32 MB

**Table 3.** Benefits of advanced sensing in smart cities.

	**Without Advanced Sensing**	**With Advanced Sensing**
**Structural Health Monitoring**	- High costs due to the number of personnel required for scheduled inspections - Visual Inspection is not always effective	- Autonomous monitoring system reduces costs of scheduled inspections and provides continuous monitoring - Enables a more accurate analysis of the structure's state than visual inspection
**Water Distribution**	- High costs in disasters caused by missed or late leak detections	- Enables mitigation of costs caused by possible accidents due to late leak detections - Monitoring the quality of water ensures that the water is safe for human consumption
	**Without Advanced Sensing**	**With Advanced Sensing**
**Electricity Distribution**	- Inaccurate metering and demand prediction	- Advanced energy sensing enables more accurate metering and demand prediction
**Smart Buildings**	- High electricity and water consumption	- Reduction in water and electricity consumption due to HVAC and light control
**Intelligent Transportation**	- Inefficient traffic control schemes causing traffic jams	- Improved traffic control schemes which are adaptive to traffic conditions
**Surveillance**	- Need for a human operator who is prone to distraction	- Intelligent detection of abnormal situations without the need of an operator
**Environmental Monitoring**	- Hazardous conditions, like presence of dangerous gases, maybe detected too late	- Continuous environmental gas sensing ensures that hazardous conditions can be detected timely

**Table 4.** Current smart city pilot projects.

**Location**	**Aim**
Songdo, Korea	Ubiquitous sensing, Fully automated buildings, smart street lighting, smart meters and tele-presence
PlanIT Valley, Portugal	Ubiquitous sensing, plan to deploy 100,000,000 sensors
Fujisawa SST, Japan	Ubiquitous sensing, plan to reduce carbon footprint by 70%
Amsterdam, Netherlands	Smart energy management systems, smart grids and other energy efficiency initiatives
Groening, Netherlands	Improved public transportation systems with real-time access to location and schedule
Norfolk, England	Improved data delivery services for the community, improved data collection and analysis systems for the municipality
Santander, Spain	Smart parking systems, participatory sensing and environmental monitoring
Barcelona, Spain	Smart garbage collection, smart parking systems and smart street lighting
Malaga, Spain	Reduction of carbon dioxide emissions by 6,000 tons per annum
Vienna, Austria	Improvement in energy efficiency and climate protection; reduction in carbon footprint
